# Riboflavin Plays a Pivotal Role in the UVA-Induced Cytotoxicity of Fibroblasts as a Key Molecule in the Production of H_2_O_2_ by UVA Radiation in Collaboration with Amino Acids and Vitamins

**DOI:** 10.3390/ijms21020554

**Published:** 2020-01-15

**Authors:** Satoshi Yoshimoto, Nana Kohara, Natsu Sato, Hideya Ando, Masamitsu Ichihashi

**Affiliations:** 1 Department of Applied Chemistry and Biotechnology, Okayama University of Science, Okayama 700-0005, Japan; t17sd03ys@ous.jp (S.Y.); ando@dac.ous.ac.jp (H.A.); 2 Anti-Aging Medical Research Center, Doshisha University, Kyoto 610-0394, Japan; 3 Faculty of Pharmaceutical Science, Kobe Gakuin University, Kobe 650-8586, Japan; 4 Arts Ginza Clinic, Tokyo 105-0004, Japan

**Keywords:** UVA, photosensitization, fibroblast, hydrogen peroxide, superoxide, singlet oxygen, photo-aging

## Abstract

To investigate environmental factors that contribute to ultraviolet A (UVA)-induced oxidative stress, which accelerates the senescence and toxicity of skin cells, we irradiated human fibroblasts cultured in commonly used essential media with UVA and evaluated their viability and production of reactive oxygen species. The viability of fibroblasts exposed to a single dose of 3.6 J/cm^2^ UVA was not reduced when cultured in Hanks balanced salt solution, but it was significantly decreased when cultured in Dulbecco’s modified Eagle’s medium (DMEM), which contains various amino acids and vitamins. Furthermore, cell viability was not reduced when fibroblasts were cultured in DMEM and treated with a hydrogen peroxide (H_2_O_2_) scavenger such as glutathione or catalase added after UVA irradiation. In addition, we confirmed that the production of H_2_O_2_ was dramatically increased by UVA photosensitization when riboflavin (R) coexisted with amino acids such as tryptophan (T), and found that R with folic acid (F) produced high levels of H_2_O_2_ after UVA irradiation. Furthermore, we noticed that R and F or R and T have different photosensitization mechanisms since NaN_3_, which is a singlet oxygen quencher, suppressed only R and T photosensitization. Lastly, we examined the effects of antioxidants (L-ascorbic acid, trolox, L-cysteine, and L-histidine), which are singlet oxygen or superoxide or H_2_O_2_ scavengers, on R and F or on R and T photosensitization, and found that 1 mM ascorbic acid, Trolox, and L-histidine were strongly photosensitized with R, and produced significant levels of H_2_O_2_ during UVA exposure. However, 1 mM L-cysteine dramatically suppressed H_2_O_2_ production by UVA photosensitization. These data suggest that a low concentration of R-derived photosensitization is elicited by different mechanisms depending on the coexisting vitamins and amino acids, and regulates cellular oxidative stress by producing H_2_O_2_ during UVA exposure.

## 1. Introduction

Deleterious damage of the skin such as photo-aging and cancers that are caused by chronic long-term exposure to solar ultraviolet (UV) radiation have been extensively documented by a large number of basic and clinical studies [1,2,3,4]. Therefore, the prevention of photo-aging and skin cancers is extremely important for aging subjects in developed countries to maintain their youthful and healthy skin complexion, since solar lentigines and skin cancers commonly develop in Asians around 20 years of age and in Caucasians after middle age, respectively [5].

The UV spectrum is divided into three groups based on the wavelength: ultraviolet C (UVC: 100–280 nm), ultraviolet B (UVB: 280–320 nm), and ultraviolet A (UVA: 320–400 nm) [6]. UVA is further subdivided into UVA2 (320–340 nm) and UVA1 (340–400 nm) [7]. Solar UV radiation at the Earth’s surface is approximately 90–99% UVA and 1–10% UVB. UVC is almost completely absorbed by the ozone layer in the atmosphere and reaches the surface of the earth at a negligible dose, except for the tops of high mountains, such as Mt. Everest. UVC radiation induces typical DNA damage, cyclobutane pyrimidine dimers in the nuclear DNA of cells, and also produces erythema in human skin [8]. UVB is absorbed by DNA, RNA, and amino acids within and outside of cells in the skin and eyes [9,10,11]. UVB has been shown to be the main cause of skin responses to acute inflammation and also for chronic solar radiation-induced photo-aging and skin cancer formation [12,13]. In contrast, UVA does not induce an acute skin response called a sunburn, but contributes to the production of chronically-induced deleterious types of skin damage that result in photo-aging, especially wrinkles that are associated with histopathological actinic elastosis and pigmented spots known as solar lentigines [14]. The mechanism of UVA-induced chronic skin damage has been reported to be caused by reactive oxygen species (ROS) produced by the transfer of photo-activated electrons and energy of chemicals, such as flavins, tryptophan-derived 6-formylindolo[3,2-b]carbazole (FICZ), porphyrins, and melanin [15,16,17]. UVA induces photo-damage through type I (electron transfer), major type Ⅱ (singlet oxygen: energy transfer), and minor type Ⅱ (superoxide: electron transfer) mechanisms. The type I mechanism does not require oxygen for the induction of photo-damage, whereas type Ⅱ mechanisms proceed only in the presence of oxygen [18]. We recently reported that chronic low dose UVA radiation induces the cellular senescence of fibroblasts in vitro at a total dose of 36 J/cm^2^ within two weeks, and suggested a pivotal role for hydrogen peroxide (H_2_O_2_) in the induction of cell senescence, which is produced by UVA absorption and possibly by riboflavin, known as a potent UVA chromophore in the medium [19].

In the present study, we aimed to clarify the mechanism involved in the production of H_2_O_2_ by UVA radiation in vitro in the presence or absence of several UVA photosensitizers, amino acids, and vitamins. We initially confirmed the role of riboflavin, vitamin B_2_, which is well-known as a photosensitizer [20] in the formation of H_2_O_2_ in Hanks Balanced Salt Solution (HBSS) exposed to UVA radiation. The results showed that riboflavin alone in HBSS produced an extremely small amount of H_2_O_2_, but riboflavin coexisting with other chemicals, such as tryptophan, tyrosine, or folic acid, produced high levels of H_2_O_2_ in the medium after UVA irradiation at a single dose of 3.6 J/cm^2^. We then used medium containing all kinds of amino acids and only one vitamin each in Dulbecco’s modified Eagle’s medium (DMEM), and confirmed that riboflavin is a major vitamin that produces H_2_O_2_ during UVA exposure. Additionally, we confirmed that tryptophan and tyrosine are major amino acids responsible for the production of H_2_O_2_ in the presence of riboflavin. We considered that a singlet oxygen, which is one of the other types of ROS produced by UVA, is an early important type of ROS leading to H_2_O_2_ formation, and we showed that NaN_3_, which is a potent singlet oxygen scavenger [21], suppressed the formation of H_2_O_2_ in medium containing riboflavin and tryptophan. Furthermore, we found that the production of H_2_O_2_ in medium containing riboflavin and folic acid was not suppressed by NaN_3_, which suggests a role for minor type Ⅱ (superoxide) and type I photosensitization in the formation of H_2_O_2_. These results indicate that riboflavin, which is present at a low concentration in the medium, may trigger UVA-induced photoreactions by absorbing UVA photon energy, generating triplet states of riboflavin, and transferring its energy to tryptophan and folic acid in the formation of singlet oxygen and/or superoxide, which leads to the production of H_2_O_2_ and is toxic to cells. In addition, we investigated the effects of L-ascorbic acid, trolox, L-cysteine, and L-histidine, which are well known ROS scavengers [22,23,24,25], in the production of H_2_O_2_ by UVA irradiation. The results showed that the addition of ascorbic acid and L-histidine to riboflavin and tryptophan or folic acid in the medium significantly increased the level of H_2_O_2_, but, in contrast, L-cysteine suppressed photosensitization by riboflavin. Lastly, our study suggests that the use of sunscreens that contain efficient materials to scavenge singlet oxygen, superoxide, and H_2_O_2_ and activators of antioxidant systems within cells is the most effective way to protect the skin against serious damage from solar radiation to maintain youthful and healthy skin without photo-aging and to prevent the formation of skin cancers in later years.

## 2. Results

### 2.1. H_2_O_2_ Generated by UVA in DMEM Is Highly Cytotoxic

**Figure 1:**
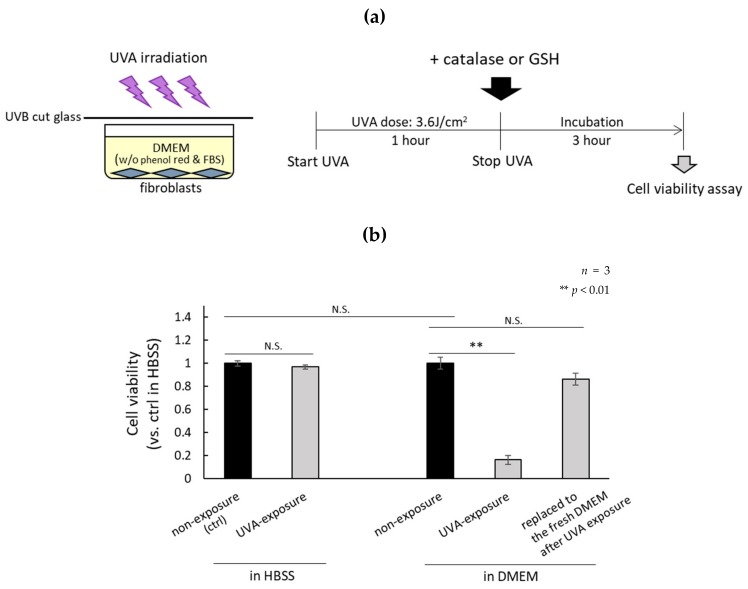

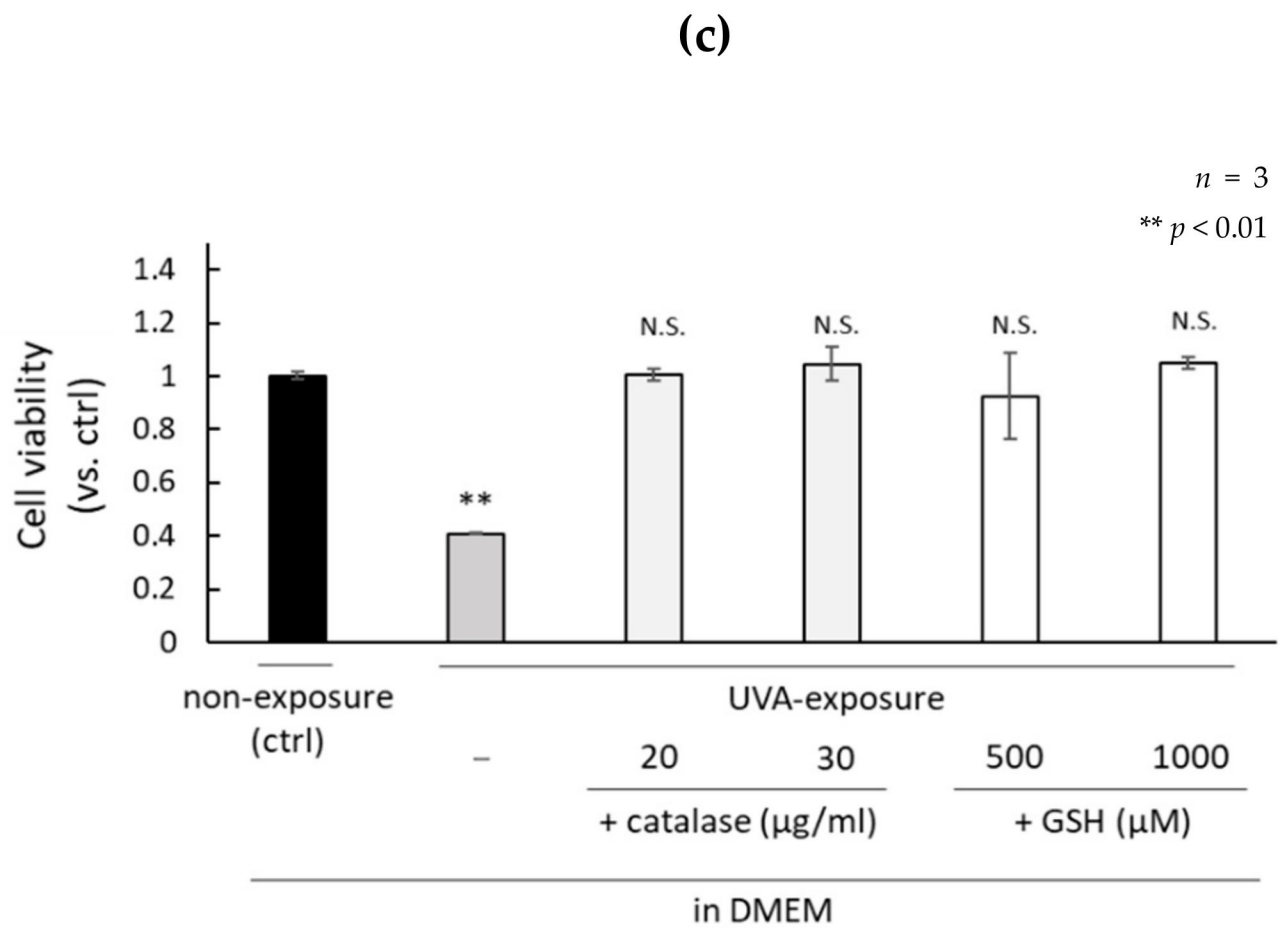
The production of H_2_O_2_ by UVA exposure decreases the cell viability of normal human dermal fibroblasts. (**a**) Normal human dermal fibroblasts were irradiated with 1.0 mW/cm^2^ UVA for 1 h under various conditions and cell viability was measured 3 h after UVA irradiation. (**b**) Cell viabilities of UVA-irradiated fibroblasts cultured in DMEM (containing UVA photosensitizers) and in HBSS (without UVA photosensitizers) were compared with non-UVA irradiated fibroblasts (ctrl). (**c**) Fibroblasts cultured in DMEM were treated with catalase (20 or 30 µg/mL) or GSH (500 or 1000 µM) after UVA irradiation, and cell viability was compared with non-irradiated cells. N.S.: not significant.

The oxidative stress of fibroblasts exposed to UVA in DMEM, which contains amino acids, vitamins, glucose, and buffer components, or in HBSS, which contains only glucose and buffer-related components, were compared. UVA irradiation conditions are shown in Figure 1a. The cell viability of fibroblasts cultured in DMEM exposed to UVA decreased significantly, but remained at the same level as non-irradiated fibroblasts cultured in DMEM when the DMEM was replaced with fresh DMEM immediately after the UVA exposure (Figure 1b). We previously reported that H_2_O_2_ generated by UVA exposure of DMEM induces the senescence of fibroblasts [19]. Therefore, we studied the effects of catalase and glutathione (GSH) on the viability of UVA-irradiated fibroblasts cultured in DMEM and confirmed the preventive effects of those antioxidants when added to the DMEM prior to exposure with UVA (Figure 1c). These results indicated that the H_2_O_2_ generated in DMEM by UVA exposure is the major type of ROS involved in UVA-induced cell toxicity. 

### 2.2. Photosensitization by UVA Is Amplified in the Presence of Amino Acids and Vitamins

**Figure 2:**
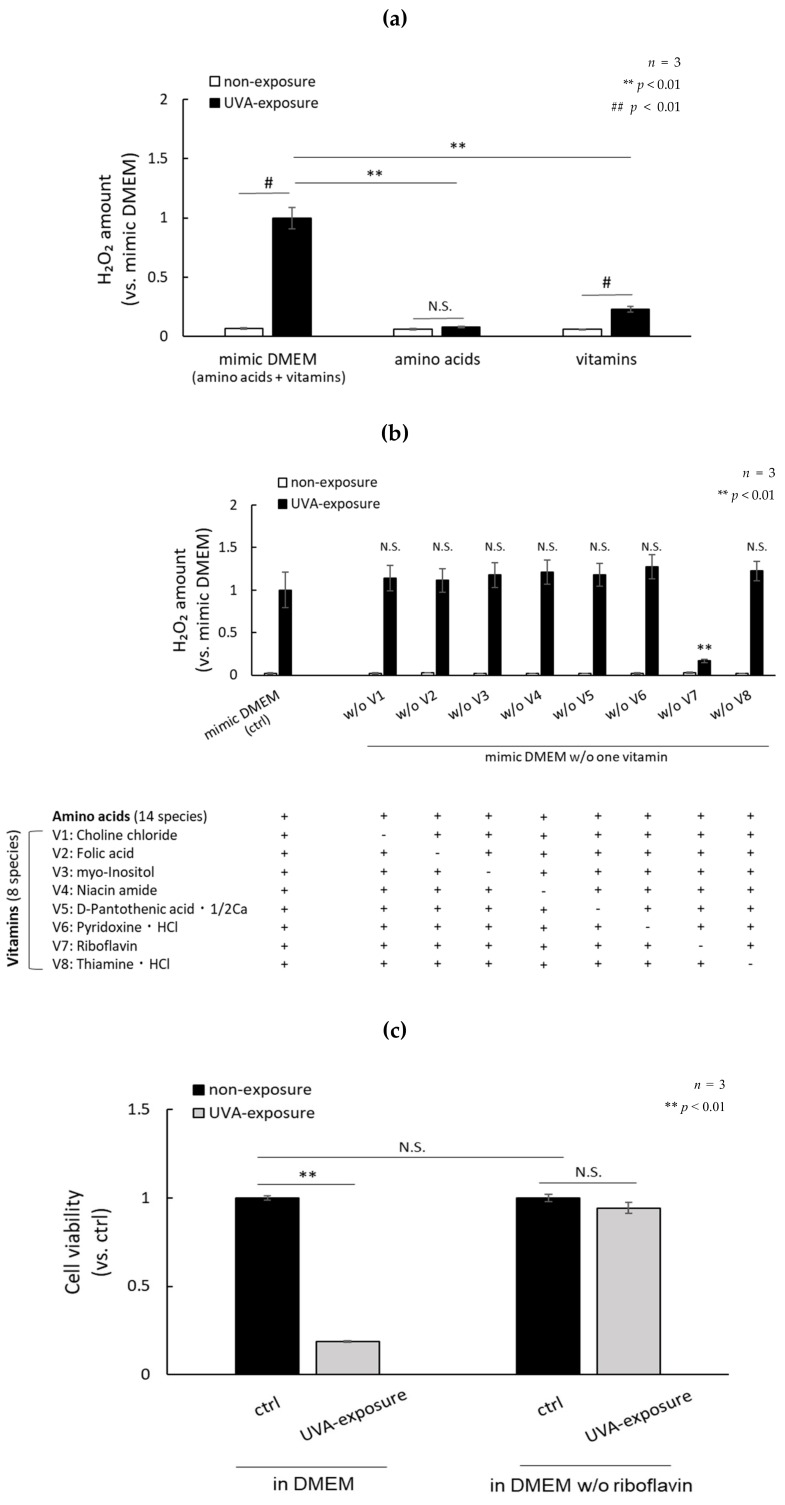

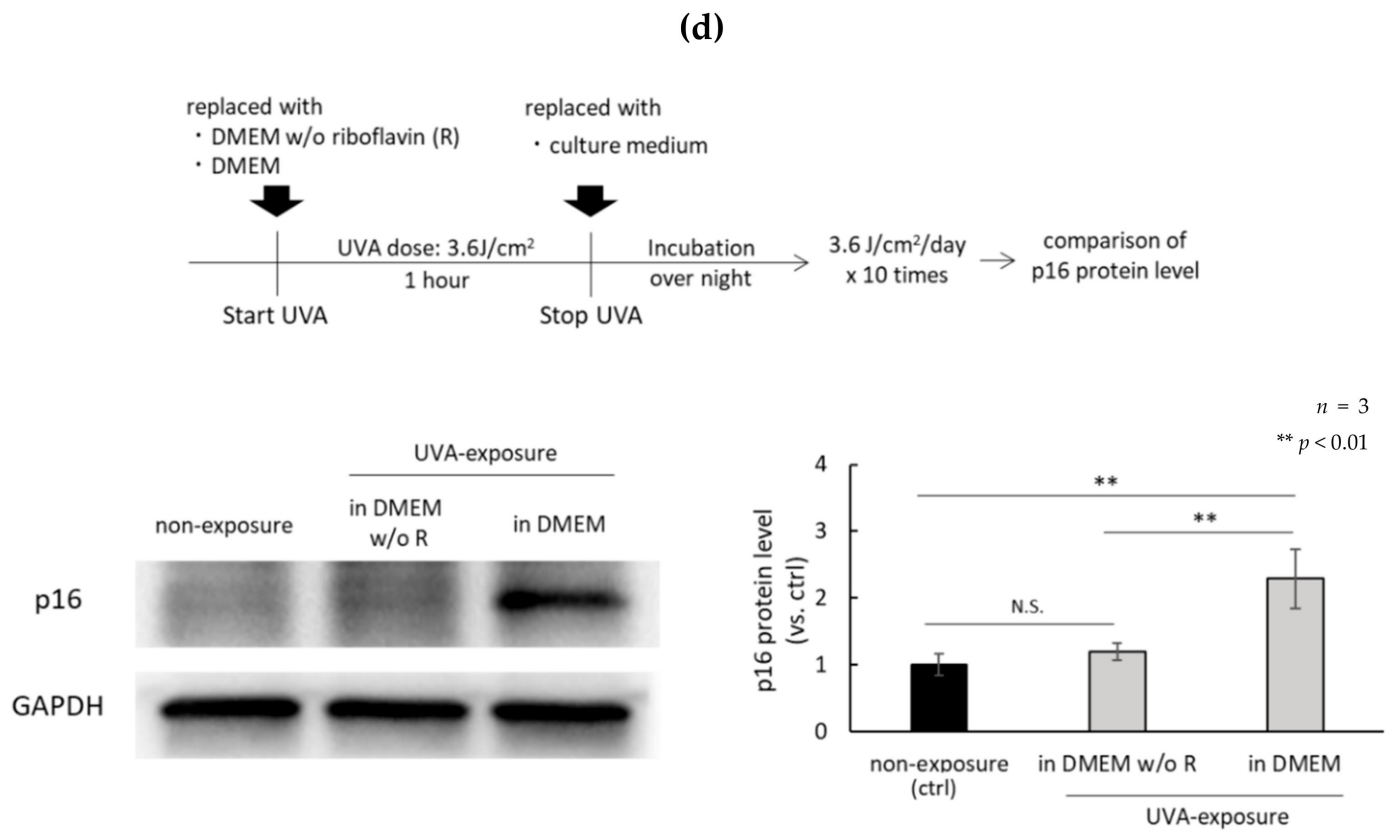
Effect of riboflavin on the production of H_2_O_2_ in DMEM after exposure to a dose of 3.6 J/cm^2^ UVA. (**a**) Media containing all 14 amino acids at the same concentration as in DMEM and containing each of the eight vitamins were irradiated with UVA at a dose of 3.6 J/cm^2^ and the production of H_2_O_2_ was evaluated. (**b**) Various kinds of DMEM free from each of the 8 vitamins were irradiated with 1.0 mW/cm^2^-UVA for 1 h and H_2_O_2_ levels were measured. DMEM without riboflavin showed an extremely low level of H_2_O_2_. (**c**) Normal human dermal fibroblasts cultured in DMEM in the presence or absence of riboflavin were irradiated with 1.0 mW/cm^2^ UVA for 1 h and cell viability was measured 3 h after the UVA irradiation. (**d**) Comparison of p16 levels of fibroblasts after UVA repeated irradiation in DMEM with or without riboflavin. [top] Protocol for UVA repeated irradiation of fibroblasts. [left] p16 content in fibroblasts after the final UVA irradiation detected by Western blotting. [right] Densitometric analysis of Western blots for p16 normalized by GAPDH.

**Figure 3:**
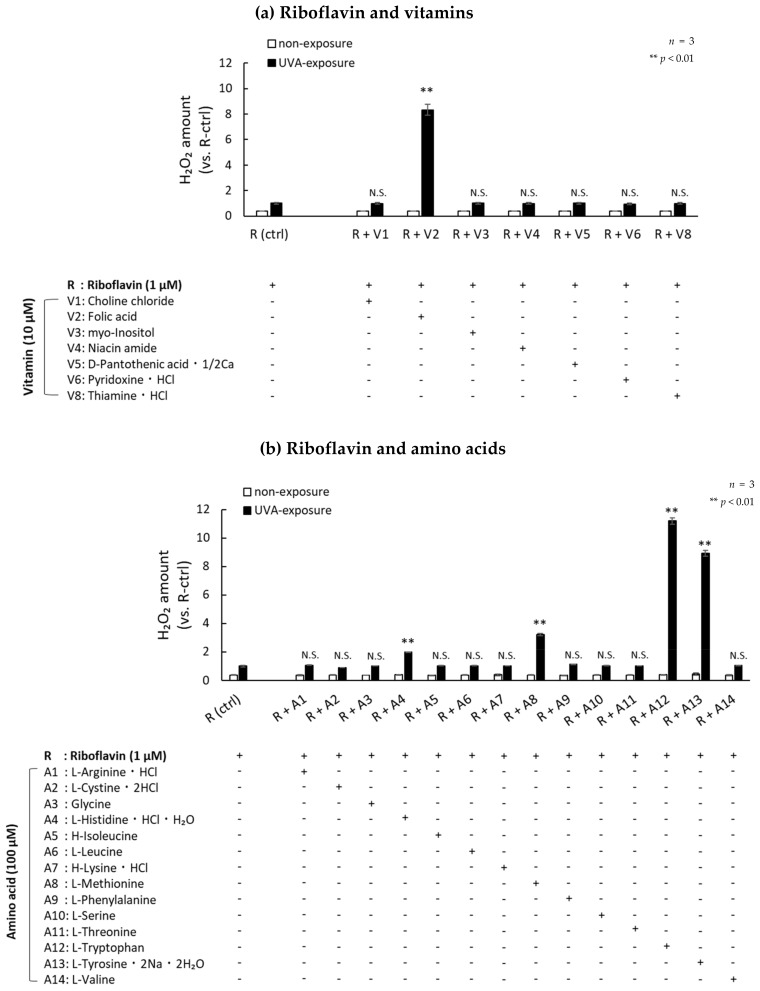
Identification of DMEM components that produce H_2_O_2_ following UVA exposure in the presence of riboflavin. HBSS medium containing riboflavin (1 µM) and one each of: (**a**) eight vitamins (each at 10 µM) or (**b**) 14 amino acids (each at 100 µM) was irradiated with 3.6 J/cm^2^ UVA. After UVA irradiation, the level of H_2_O_2_ in each HBSS condition was measured. All measurements were compared to the amount of H_2_O_2_ produced when a single riboflavin was exposed to UVA.

Chemical components of DMEM, such as folic acid and riboflavin, have been reported to produce H_2_O_2_ [26]. In order to study which vitamins and/or amino acids are deeply involved in the production of H_2_O_2_, we evaluated the production of H_2_O_2_ in HBSS containing each of the 14 amino acids and the eight vitamins contained in DMEM exposed to UVA radiation. Table 1 shows the 14 amino acids and eight vitamins contained in DMEM. We compared the amount of H_2_O_2_ generated when those components were irradiated with UVA. The amount of H_2_O_2_ did not increase significantly when UVA was exposed to a mixed solution of 14 amino acids. Exposure of a solution containing eight vitamins to UVA increased H_2_O_2_ production, but at significantly lower levels than a solution mimicking DMEM, which contained 14 amino acids and those same eight vitamins (Figure 2a). In addition, the amount of H_2_O_2_ produced in DMEM by UVA exposure was significantly reduced only when riboflavin was removed from the medium. There was no significant change in the amount of H_2_O_2_ after UVA exposure in conditions that excluded choline chloride, folic acid, myo-inositol, niacin amide, D-pantothenic acid, 1/2Ca, pyridoxine, HCl and thiamine, and HCl (Figure 2b). The cell viability of fibroblasts cultured in DMEM and exposed to UVA without riboflavin remained high (Figure 2c). The protein level of p16 in fibroblasts exposed to repetitive UVA in riboflavin-free DMEM for 10 days remained at a low level compared to fibroblasts exposed in DMEM (Figure 2d). These data indicate that riboflavin is a master regulator of the production of H_2_O_2_ by UVA photosensitization. Lastly, we clarified the components in DMEM that produce H_2_O_2_ during UVA exposure by mixing them with riboflavin. Although many components in DMEM were not involved in H_2_O_2_ production, folic acid, tryptophan, tyrosine, methionine, and histidine increased the production of H_2_O_2_ during UVA exposure in the presence of riboflavin (Figure 3).

### 2.3. H_2_O_2_ Generated by UVA Exposure Is Amplified by a Photosensitization Reaction between Riboflavin and Amino Acids via a Singlet Oxygen

**Figure 4:**
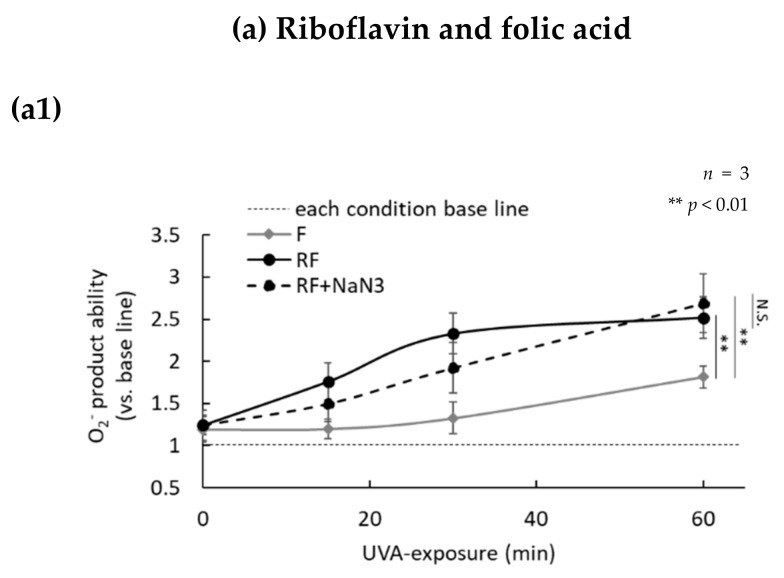

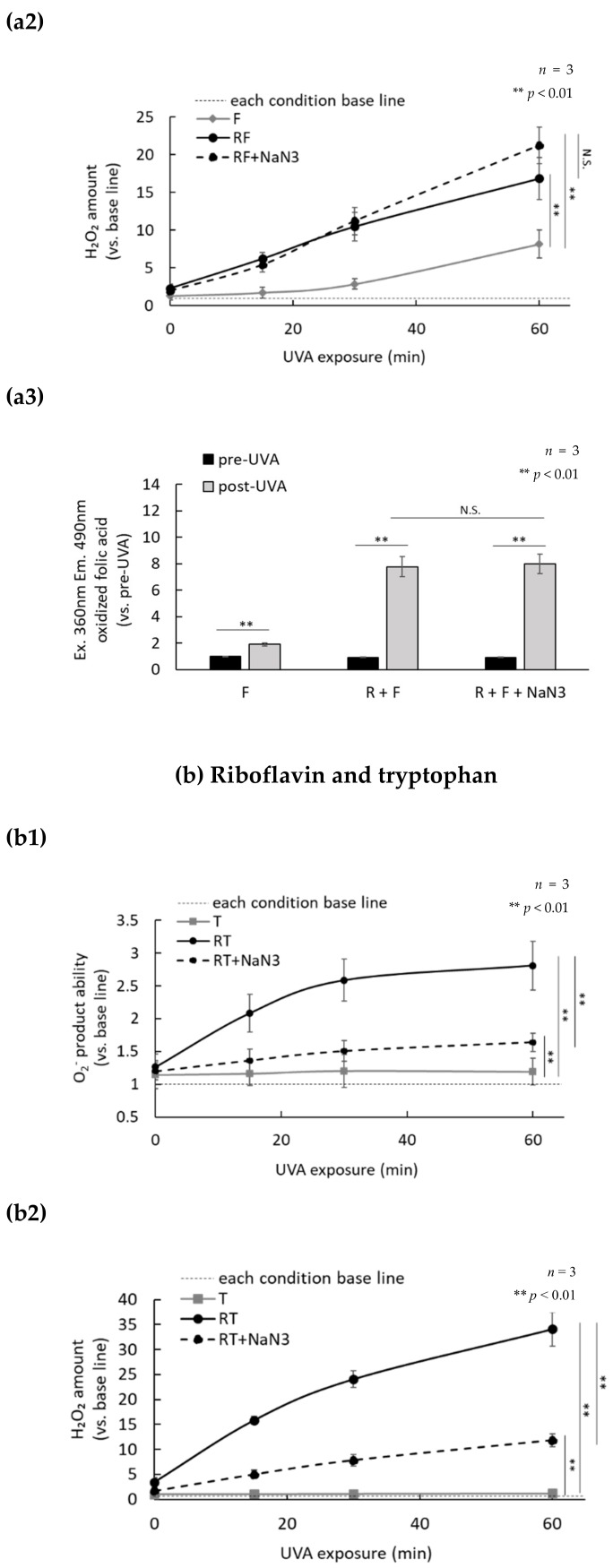

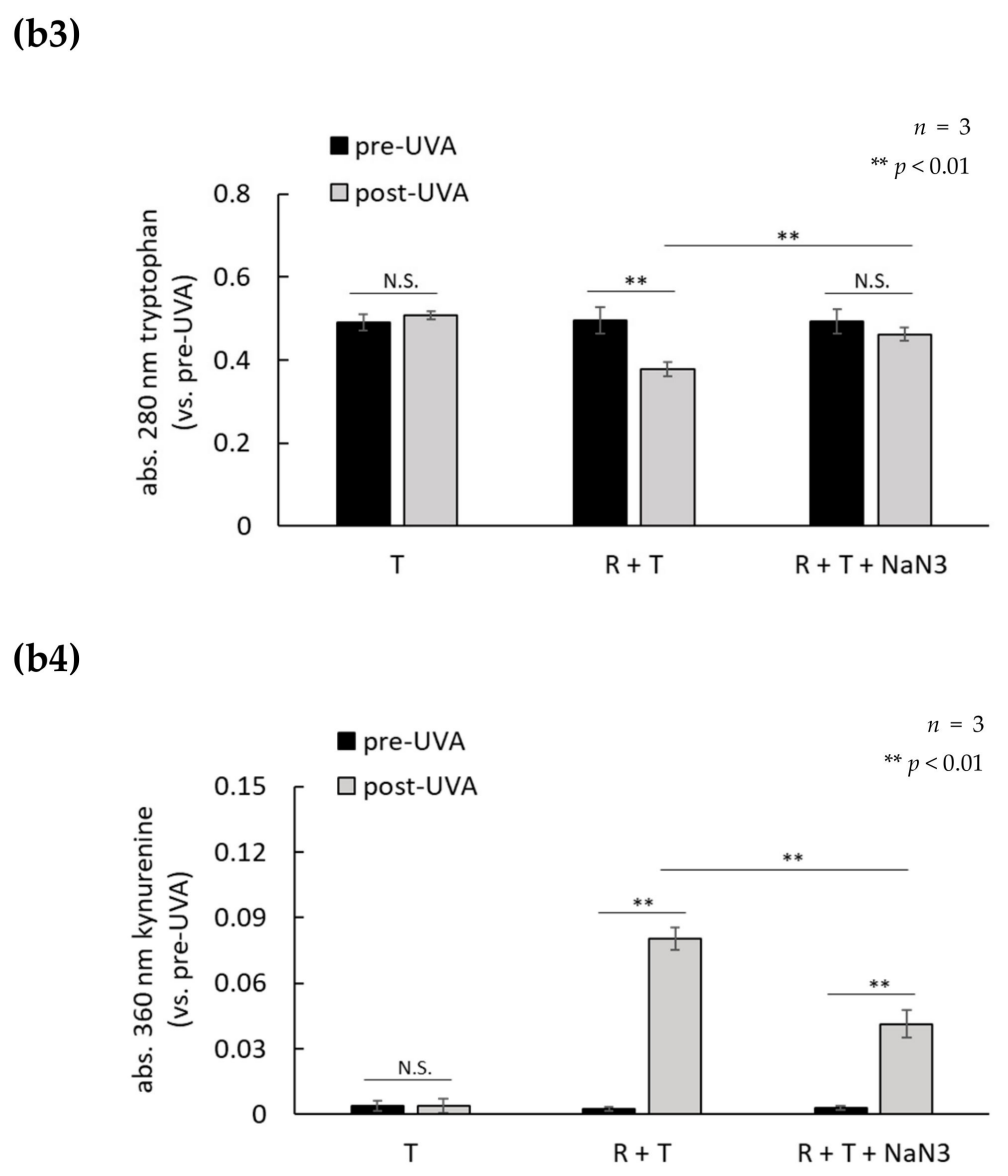
Effect of 1 mM NaN_3_ on superoxide and H_2_O_2_ production in DMEM evaluated at different times of UVA irradiation. At UVA exposure times of 0, 15, 30, and 60 min, the superoxide was measured. The levels of H_2_O_2_ were measured in the presence or absence of NaN_3_. Relative values were calculated based on each non-irradiated group. (**a1**) Superoxide production ability in medium containing riboflavin + folic acid in the absence (solid line) or presence (dotted line) of NaN_3_. (**a2**) H_2_O_2_ production curves were almost the same as those of O_2_^-^ in both the absence or presence of NaN_3_. (**a3**) Oxidized folic acid auto-fluorescence (ex. 360 nm, em. 490 nm) before and after UVA exposure. (**b1**) Superoxide production in medium containing riboflavin and tryptophan in the absence (solid line) or presence (dotted line) of NaN_3_. The production of superoxide was strongly inhibited in the presence of NaN_3_. (**b2**) H_2_O_2_ production was increased in a UVA exposure time-dependent manner but was inhibited significantly by NaN_3_. (**b3**) Comparison of tryptophan absorption (280 nm), and (**b4**) kynurenine absorption (360 nm) before and after UVA exposure.

**Figure 5:**
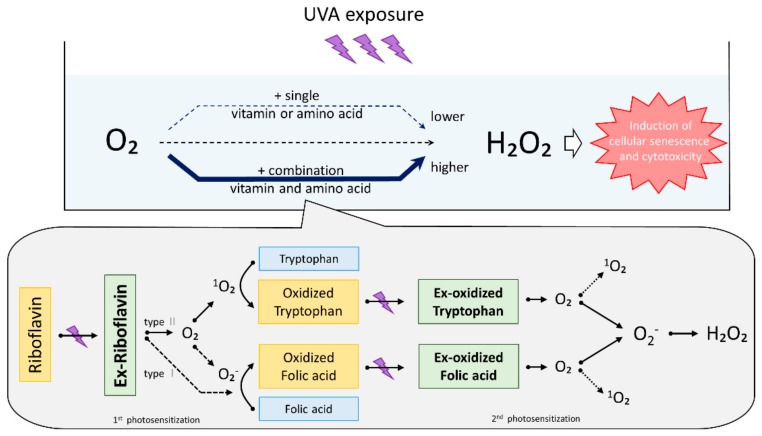
The function of H_2_O_2_ production derived from photosensitized reactions with folic acid or tryptophan using riboflavin as an initiator. Amino acids and vitamins were oxidized by type I or type Ⅱ photosensitization by excited riboflavin during UVA exposure. Lastly, oxidized-tryptophan and oxidized-folic acid act to absorb UVA radiation as secondary UVA photosensitizers and promote the production of singlet oxygen and superoxide, which leads to high levels of H_2_O_2_ formation.

Riboflavin is known to generate superoxide and singlet oxygen during UVA-induced photosensitization. In order to study the role of photosensitization in the UVA-induced production of H_2_O_2_, the production of superoxide and H_2_O_2_ was measured in the presence or absence of NaN_3_, which is a singlet oxygen scavenger. Superoxide production in medium containing riboflavin alone was not increased by UVA irradiation, but the production of superoxide in medium with folic acid alone began to increase slightly after 30 min of UVA exposure (Figure 4a). Furthermore, superoxide was produced at a high-level during UVA exposure in medium containing both riboflavin and folic acid, and reached approximately 2.5 times that level in medium containing folic acid alone 60 min after UVA exposure. In addition, the production of superoxide in medium containing both riboflavin and folic acid was not suppressed by the addition of NaN_3_. Furthermore, the production of H_2_O_2_ was also increased similarly to the pattern of superoxide production (Figure 4(a1,a2)). In addition, we measured the auto-fluorescence of folic acid since oxidized folic acid is known to auto-fluorescence [26]. Compared to conditions when a single folic acid was exposed to UVA, the auto-fluorescence of UVA-exposed folic acid in the presence of riboflavin was significantly increased. That increase in auto-fluorescence was not suppressed by the addition of NaN_3_ (Figure 4(a3)). The medium containing tryptophan alone exposed to UVA did not produce superoxide or H_2_O_2_, but did produce superoxide and H_2_O_2_ immediately after a single dose of UVA irradiation in the presence of riboflavin. The UVA-induced production of superoxide and H_2_O_2_ in medium containing both riboflavin and tryptophan was strongly suppressed by the addition of NaN_3_, which suggests that singlet oxygen is involved in the photosensitization between riboflavin and tryptophan (Figure 4b). Those results suggest that the photosensitization mechanism of riboflavin-folic acid is different from that of riboflavin-tryptophan. Since kynurenine, which is an oxidative degradation product of tryptophan, is known to produce H_2_O_2_ after UVA exposure [27], we measured the levels of tryptophan and kynurenine in medium containing both riboflavin and tryptophan, using their characteristic light absorbance at 280 nm and 360 nm, respectively. We observed that the oxidative degradation of tryptophan and kynurenine production in medium increased by UVA exposure in the presence of only riboflavin, and that degradation and production was suppressed by NaN_3_ added to the medium immediately before the UVA irradiation (Figure 4(b3,b4)). These data showed that amino acids and vitamins are oxidized by UVA photosensitization (type I or type Ⅱ) of riboflavin as an initial reaction, and that H_2_O_2_ is produced at a high concentration by the UVA photosensitization of oxidized amino acids and vitamins (Figure 5).

### 2.4. UVA Exposure to Antioxidants in the Presence of Riboflavin Increases the Production of H_2_O_2_

**Figure 6:**
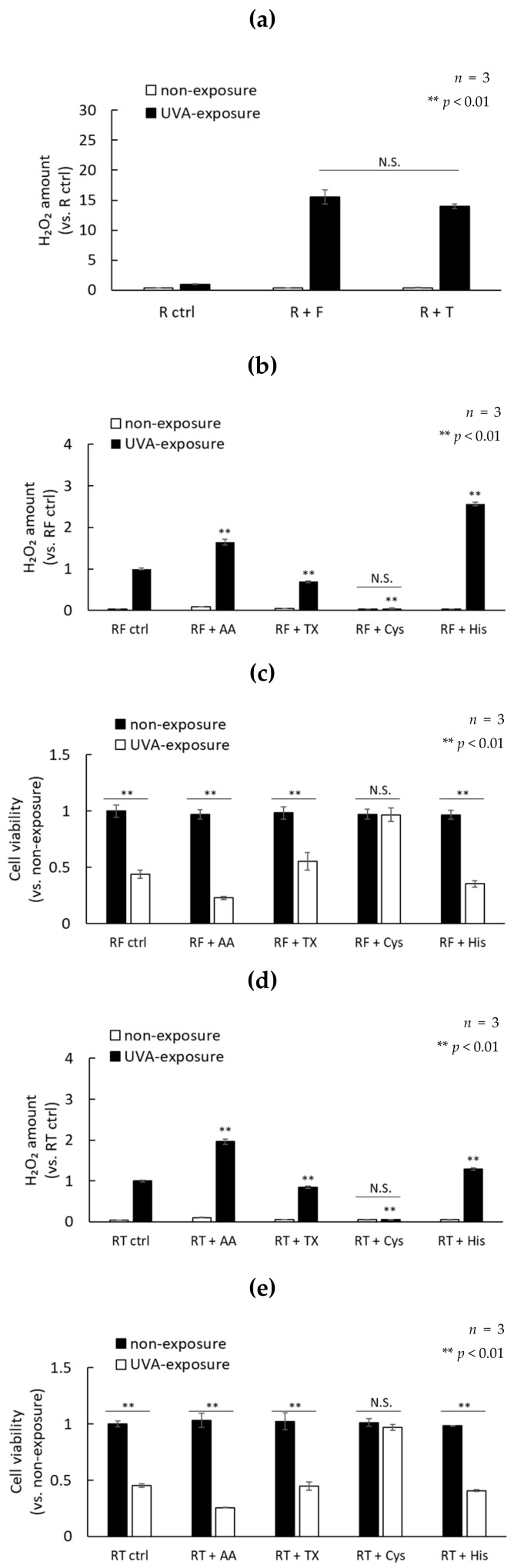
The effects of antioxidants on photosensitization between riboflavin and folic acid (R and F) or riboflavin and tryptophan (R and T) were evaluated. (**a**) The production of H_2_O_2_ in the presence of 30 µM folic acid or 100 µM tryptophan irradiated with 3.6 J/cm^2^ UVA in the presence of 1 µM riboflavin. (**b**) The production of H_2_O_2_ and (**c**) cell viability after irradiation with 3.6 J/cm^2^ UVA in the presence of a mixed solution of 1 µM riboflavin, 30 µM folic acid, and 1 mM antioxidants. (**d**) The production of H_2_O_2_ and (**e**) cell viability after irradiation with 3.6 J/cm^2^ UVA in the presence of a mixed solution of 1 µM riboflavin, 100 µM tryptophan, and 1 mM antioxidants.

These results clarified that a singlet oxygen and the superoxide are involved in the production of H_2_O_2_ by UVA photosensitization using riboflavin as an initiator. Therefore, we examined whether treatment with antioxidants could reduce cell damage caused by UVA. Ascorbic acid, trolox (water-soluble vitamin E), L-histidine, and L-cysteine were used as antioxidants. It has been reported that ascorbic acid and trolox have radical scavenging abilities. L-histidine has a singlet oxygen scavenging ability and L-cysteine has a high H_2_O_2_ scavenging ability [28]. Therefore, the effects of those antioxidants on photosensitization between riboflavin and folic acid (R and F) or riboflavin and tryptophan (R and T) were evaluated. The concentrations of folic acid and tryptophan were determined so that the amounts of H_2_O_2_ after UVA exposure are comparable (Figure 6a). The production of H_2_O_2_ by the photosensitization of R and F or R and T was completely suppressed by the addition of 1 mM cysteine, and cell viability was not reduced. Furthermore, 1 mM trolox showed a tendency to suppress H_2_O_2_ production and cell viability was decreased. In contrast, the production of H_2_O_2_ by photosensitization (in particular R and F) was significantly increased by the addition of 1 mM histidine, which causes strong cytotoxicity. Ascorbic acid showed results similar to histidine (Figure 6b–e).

**Figure 7:**
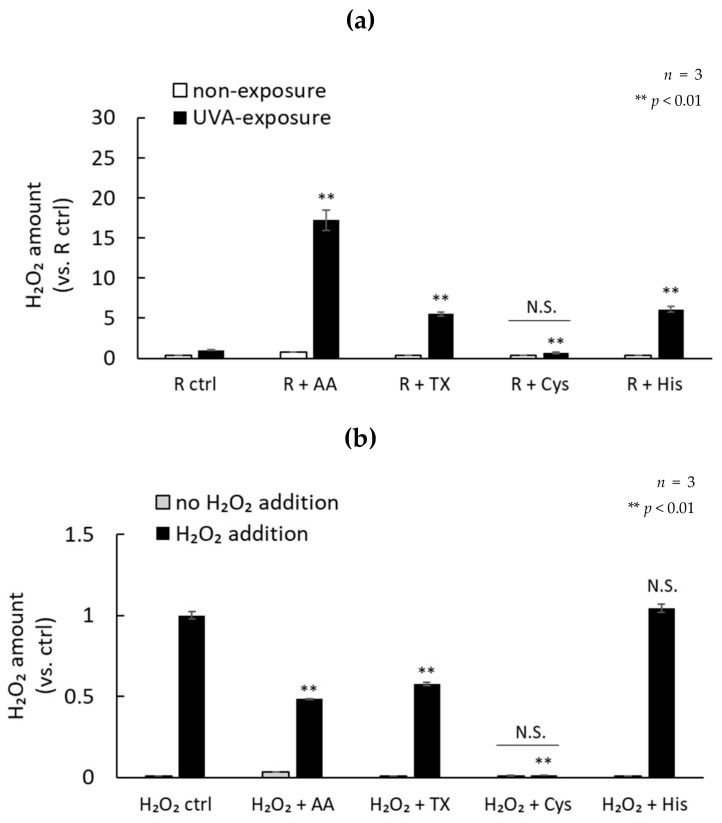
Comparison of H_2_O_2_ production by antioxidants exposed to UVA radiation. (**a**) The production of H_2_O_2_ by UVA exposure in the presence of 1 mM antioxidants and 1 µM riboflavin. (**b**) The amount of H_2_O_2_ remaining 15 min after adding 1 mM antioxidant to 100 µM H_2_O_2_ solution.

Lastly, we measured the photosensitization of each antioxidant and riboflavin, and the H_2_O_2_ scavenging ability. Each antioxidant other than cysteine in the presence of riboflavin enhanced H_2_O_2_ production by UVA irradiation. In particular, ascorbic acid showed a remarkably enhanced production of H_2_O_2_. The addition of cysteine to riboflavin reduced the production of H_2_O_2_ after UVA exposure to a similar level as non-exposure (Figure 7a). L-cysteine effectively scavenged 100 µM H_2_O_2_. In addition, ascorbic acid and trolox also showed high H_2_O_2_ scavenging ability, but L-histidine did not scavenge H_2_O_2_ (Figure 7b). These data suggest that photosensitized antioxidants may contribute to the induction of oxidative damage by UVA exposure in the presence of riboflavin. Furthermore, our data suggest that chemicals with a high H_2_O_2_ scavenging ability, such as L-cysteine, may be highly effective in reducing the cytotoxicity caused by UVA.

## 3. Discussion

We and other research groups have demonstrated that chronic repetitive UVA radiation as well as a single high dose of UVA radiation causes cellular senescence in vitro and contributes to the induction of skin photo-aging in vivo, mostly by the generation of ROS [19,29], particularly H_2_O_2_, which has a long half-life compared with O_2_^−^ and ^1^O_2_. To induce biological reactions in the skin, UVA radiation is absorbed by molecules, called endogenous and exogenous chromophores, such as flavins, porphyrins, pterins, urocanic acid, FICZ, heme-oxygenase-1, and NADPH-oxidase. Those chromophores are known to acquire reactive long-lived triplet states and transfer their energy to oxygen molecules to yield ROS and/or electron-transfer reactions to other molecules including genomic and mitochondrial DNA, which lead to physiological and pathological skin changes [30]. Singlet oxygen has been postulated to be an initial type of ROS involved in UVA-induced biological reactions by showing the involvement of singlet oxygen in the enhanced expression of genes encoding heme oxygenase and collagenase by indirect studies using singlet oxygen scavengers, hydroxy radical scavengers, deuterium oxide, and mannitol dimethyl sulfoxide [31].

Previously, we reported a cellular senescence model produced by the repeated irradiation of cultured human dermal fibroblasts with low-dose UVA, and showed an important role for H_2_O_2_ in UVA-induced cellular senescence. We also found that palladium and platinum nanoparticles with very strong catalase activity significantly inhibited the H_2_O_2_-induced cellular senescence. However, our previous study did not address the mechanism of H_2_O_2_ production or the roles of other types of ROS. The purpose of this study was to clarify the production mechanism of H_2_O_2_ produced by UVA exposure of commonly used essential media. At first, we showed that, among the various types of ROS, H_2_O_2_ is the main cause of the cytotoxicity induced by UVA exposure of fibroblasts cultured in DMEM. In addition, we found that H_2_O_2_ generated in DMEM exposed to UVA was dramatically amplified by the cooperative photosensitization of riboflavin with amino acids and other vitamins. Furthermore, the results indicated that there are at least two mechanisms involved in the production of H_2_O_2_, superoxide-mediated, and singlet-oxygen-mediated mechanisms initially produced by the photosensitization of riboflavin exposed to UVA radiation.

One type of photosensitization occurs in medium containing riboflavin and folic acid. Folic acid in aqueous solution easily undergoes oxidative degradation upon UVA exposure to produce a fluorescent pterin derivative [32]. That pterin derivative is known to produce superoxide, H_2_O_2_, and other types of ROS by absorbing UVA [33]. In addition, pterin derivatives are reported to accumulate in vitiligo skin [34,35], and to induce ultraviolet stress in melanocytes. When medium containing both folic acid and riboflavin was exposed to UVA, blue fluorescence derived from pterin derivatives appeared in the medium earlier than in medium containing folic acid alone. Those results indicate that the oxidative degradation of folic acid proceeds only very slowly in HBSS containing folic acid alone, but occurs rapidly in the presence of the photosensitizer riboflavin. Since this reaction was not inhibited by NaN_3_, a singlet oxygen scavenger, it was thought that the oxidative degradation of folic acid was possibly promoted via photosensitization of type I (direct electron transfer) and/or minor type Ⅱ (superoxide) generated by riboflavin photosensitization.

The other type of photosensitization occurs between riboflavin and tryptophan. Since tryptophan has a maximum absorption wavelength in the UVB region (especially at 280 nm), exposure to UVB is known to produce tryptophan oxides such as FICZ and kynurenine derivatives. These tryptophan oxides have absorption wavelengths in the UVA region, and it has been reported that UVA exposure produces superoxide, H_2_O_2_, and other types of ROS [36,37,38]. Since tryptophan does not have a UVA absorption wavelength, UVA exposure of tryptophan alone did not cause the oxidative degradation of tryptophan and did not produce kynurenine. However, in our study, exposure to UVA in the presence of riboflavin decreased the 280-nm absorption by tryptophan and increased the 360-nm absorption by kynurenine. Those results indicate that the oxidative degradation of tryptophan, which does not occur in medium with tryptophan alone, may be initiated by the photosensitization of riboflavin. This phenomenon was markedly suppressed by the addition of NaN_3_, which suggests that the oxidative degradation of tryptophan may be promoted by a singlet oxygen generated at an earlier time by the photosensitization of riboflavin.

These results suggest that H_2_O_2_ produced in DMEM by UVA exposure is dramatically amplified by the following reactions. (1) The photosensitization of type I and type Ⅱ reactions by riboflavin as an initiator oxidizes tryptophan or folic acid present in the medium, (2) photo-activated tryptophan or folic acid oxidizes O_2_ molecules to produce superoxide or singlet oxygen, and, lastly, (3) H_2_O_2_ is generated by the disproportionation of superoxide anions. These reactions that occur in DMEM suggest that a low concentration of photosensitizing molecules may cause high photosensitizing reactions in human skin.

Lastly, the effects of antioxidants on the photosensitization between riboflavin-folic acid and riboflavin-tryptophan were evaluated. The results show that L-cysteine dramatically suppressed the production of H_2_O_2_ by UVA-photosensitization. However, ascorbic acid, Trolox, and L-histidine were UVA-photosensitized by riboflavin, even though they have been reported to scavenge a singlet oxygen and superoxide. In addition, it has been reported that ascorbic acid reacts with a singlet oxygen to produce H_2_O_2_ and does not reduce the damage caused by UVA exposure of albino hairless mice [39,40]. Taking these findings into consideration, we conclude that antioxidants that are strongly photosensitized with riboflavin may induce oxidative stress during UVA exposure. The concentration of riboflavin, tryptophan, and folic acid in human blood or serum has been reported as follows: Riboflavin: about 1–300 ng/mL [41,42], folic acid: about 13–57 ng/mL [43,44], and tryptophan: about 12 µg/mL [45]. Those concentrations of riboflavin and tryptophan are similar to their concentrations in DMEM, but the folic acid concentration was high compared to its concentration in DMEM. These studies suggest that UVA photosensitization in vivo may proceed via reactions between riboflavin and tryptophan and other photosensitizers and oxygen molecules. We could not find any published reports that measured the concentrations of riboflavin or tryptophan in human skin. 

In future investigations, we will try to identify antioxidants that have the ability to scavenge a singlet oxygen and superoxide without photosensitization by photosensitizers such as riboflavin. Furthermore, since this study was performed using DMEM, it will be necessary to analyze more complicated photosensitizing mechanisms in living skin that contains many amino acids and vitamins that are not in DMEM and many photosensitizers such as riboflavin.

## 4. Materials and Methods 

### 4.1. Cell Culture

Normal human dermal fibroblasts (cultured from the dermis of darkly pigmented newborn foreskins, Cascade Biologics, Portland, OR) were cultured in DMEM (Sigma, St Louis, MO, USA) supplemented with 10% normal fetal bovine serum (FBS, Hyclone Laboratories Inc, Logan, UT, USA) and 1% antibiotic/antimycotic solution (Sigma) at 37 °C in a humidified atmosphere containing 5% CO_2_. Fibroblasts with PDL less than 10 were used in these experiments.

### 4.2. UVA Irradiation

The UVA irradiation protocol has been described previously [19]. Prior to UVA irradiation, the culture medium was removed, and after washing twice with PBS, the culture medium was replaced with a sample buffer solution in each experiment after UVA irradiation. A lamp (Toshiba Lighting & Technology Corporation, Yokosuka, Japan) emitting a UVA spectrum (340–410 nm) was used as the UVA source and was adjusted to an intensity of 1.0 mW/cm^2^. Fibroblasts were irradiated for 1 h in an ice box for temperature control (3.6 J/cm^2^).

### 4.3. Cell Viability Assay

Cells were seeded in 24-well culture plates at a density of 4 × 10^4^ cells/well and were incubated for 24 h at 37 °C and 5% CO_2_. The protocol used to measure cell viability was based on the CellTiter 96^®^ Aqueous One Solution Reagent (Promega, Madison, WI, USA). After UVA irradiation, the cells were incubated for 3 h at 37 °C in a CO_2_ incubator. Afterward, the medium was changed to DMEM with 10% FBS and 1% antibiotic/antimycotic solution and 2% CellTiter 96^®^ Aqueous One Solution Reagent, and incubated further for 1 h. After the culture supernatants were transferred to 96-well plates, their absorbance was measured at 490 nm.

### 4.4. Preparation of Media Similar to DMEM

Modified mixtures of DMEM were prepared by adding selected components from the 14 amino acids and the eight vitamins contained in basic DMEM into HBSS (pH 7). The concentration of each component is as follows: Amino acids: 0.084 g/L L-Arginine HCl (Tokyo Chemical Industry Co., Ltd. (TCI), Japan), 0.0626 g/L L-Cystine diHCl (TCI), 0.03 g/L Glycine (FUJIFILM Wako Pure Chemical Corporation. (Wako), Japan) 0.042 g/L L-Histidine HCl monohydrate (TCI), 0.105 g/L L-Isoleucine (Wako), 0.105 g/L L-Leucine (TCI), 0.146 g/L L-Lysine monoHCl (TCI), 0.03 g/L Methionine (TCI), 0.066 g/L L-Phenylalanine (TCI), 0.042 g/L L-Serine (TCI), 0.095 g/L L-Threonine (TCI), 0.016 g/L L-Tryptophan (Sigma), 0.12037 g/L L-Tyrosine Disodium Salt Dihydrate (NACALAI TESQUE, INC., Japan), 0.094 g/L L-Valine (TCI). Vitamins include 0.004 g/L Choline Cl (TCI), 0.004 g/L Folic acid (Wako), 0.0072 g/L myo-Inositol (TCI), 0.004 g/L Nicotinamide (TCI), 0.004 g/L Calcium D-pantothenate (TCI), 0.00404 g/L Pyridixine HCl (Wako), 0.0004 g/L Riboflavin (Wako), and 0.004 g/L Thiamine HCl hydrate (TCI).

### 4.5. H_2_O_2_ Assay

The H_2_O_2_ assay was performed according to a previously described method [19] using 10-acetyl-3,7-dihydroxyphenoxazine (ADHP) and Horseradish peroxidase (HRP) reagents that react with H_2_O_2_ to emit fluorescence. Measurements were performed according to the instructions of the OxiSelect™ Hydrogen Peroxide/Peroxidase Assay Kit (Cell Biolabs, Inc., San Diego, CA, USA). An ADHP/HRP Working Solution was prepared by adding 100 µM ADHP and 0.2 U/mL HRP in Assay Buffer. Fifty µL UVA-exposed samples, 50 µL ADHP/HRP working solution, and 50 µL of assay buffer were transferred to 96-well plates, and the mixtures were allowed to stand at room temperature for 30 min while being protected from light. The fluorescence intensity of each mixture after incubation was measured using a microplate reader (excitation: 415 nm, emission: 610 nm).

### 4.6. Induction of Cellular Senescence by Repeated UVA Exposure 

The protocol of the induction of cellular senescence by repeated UVA exposure has been described previously [19]. Fibroblasts were seeded in 10-cm culture dishes and were washed twice with phosphate-buffered saline (PBS) and were replaced with DMEM without phenol red and FBS, and placed in boxes containing ice cubes under the dishes to keep the cells below 37 °C. Cells in dishes were irradiated with 1.0 mW cm^2^ for 1 h every day. Cells were re-cultured with fresh DMEM supplemented with 10% FBS after UVA irradiation. The UVA irradiation was repeated a total of 10 consecutive days (total dose: 36.0 J cm^2^). Cells 24 h after the last UVA irradiation were used for Western blotting.

### 4.7. Detection of p16 by Western Blotting

Detection of p16 by Western blotting was performed as previously described [19]. UVA-exposed and non-exposed control cells were solubilized in lysis buffer consisting of 0.1 M Tris-HCl, pH 7.4, containing 1% Igepal (Sigma), 0.01% Sodium lauryl sulfate (SDS: Sigma), and a complete protease inhibitor mixture (Roche Applied Science, Germany) for 30 min at 4 °C. After centrifugation (15,000 g for 5 min at 4 °C), the supernatants were used as cell extracts. Protein concentrations were measured using a BCA protein assay kit (Pierce Biotechnology, Rockford, IL, USA). The cell extracts were mixed with Laemmli sample buffer (Bio-Rad, Hercules, CA, USA), supplemented with 5% 2-mercaptoethanol, and boiled at 95 °C for 5 min. Ten µg protein from each total extract were separated by SDS-polyacrylamide gel electrophoresis on 4–20% Tris-glycine gels (Bio-Rad) and transferred electrophoretically to Polyvinylidene difluoride (PVDF) membranes (Bio-Rad). The membranes were blocked in 1x Tris Buffered Saline (TBS) with 1% Casein (Bio-Rad) for 1 h at room temperature and then incubated with primary antibodies (anti-CDKN2A/p16INK4a antibody, 1:500, Abcam, Cambridge, UK) diluted in TBS with 1% casein blocker for 1 h at room temperature. After three washes (each 5 min) with TBS-T, the blotting membranes were incubated in horseradish peroxidase–linked anti-rabbit or anti-mouse whole antibodies (1:1000; Abcam) in TBS with 1% casein blocker for 1 h at room temperature. After three washes with TBS-T, the immunoreactivities of the blots were detected using an ECL-plus Western blotting detection system (Bio-Rad), according to the manufacturer’s instructions. The BenchMark pre-stained protein ladder (Bio-Rad) was used to establish the molecular weight curve for Western blotting. For re-probing, a Re-Blot Plus Western Blot Recycling kit (Merck Millipore, Burlington, MA) was used to strip the antibodies, and the blots were restarted from the blocking step with TBS with 1% casein blocker and then recycled for another antibody (anti-GAPDH antibody, 1:1000, Abcam). The expression levels of p16 and Glyceraldehyde-3-phosphate dehydrogenase (GAPDH) were quantified by measuring the optical densities of specific bands using an image analysis system with Molecular Imager^®^ ChemiDoc^TM^ XRS+ with Image Lab^TM^ Software.

### 4.8. Superoxide Assay

The superoxide assay was carried out using a superoxide anion-2-methyl-6-methoxyphenylethynylimidazopyrazynone (MPEC) reaction kit (ATTO, Tokyo, Japan). Light emission was measured using a microplate reader. One hundred µL of each sample exposed to UVA were transferred to 96-well plates and 10 µL MPEC (300 µM stock reagent) were added, which was followed by UVA irradiation for 2 min. After UVA irradiation, the luminescence of MPEC was measured for 1 min. Readings measured at 0, 15, 30, and 60 min from the start of UVA irradiation were compared as the superoxide production ability.

### 4.9. Measurement of Auto-Fluorescence of Oxidized Folic Acid

Media containing folic acid, folic acid + riboflavin, or folic acid + riboflavin + NaN_3_ were UVA irradiated and the concentrations of those chemicals were measured before and after the UVA exposure. The auto-fluorescence intensity of oxidized folic acid was measured (excitation: 360 nm, emission: 490 nm) using a micro plate reader.

### 4.10. Measurement of Tryptophan and Kynurenine 

Tryptophan and kynurenine were measured using a variable wavelength detector at wavelengths of 280 nm and 360 nm, respectively, according to a previously published protocol [31]. Media containing tryptophan, tryptophan + riboflavin or tryptophan + riboflavin + NaN_3_, were UVA irradiated and the concentrations of those chemicals were measured before and after the UVA exposure. Absorption wavelengths were measured using a GeneQuant 1300/10 (GE Healthcare Life Science Japan, Tokyo, Japan) in a spectrofluorometric quartz cell having an optical path length of 10 mm.

### 4.11. Evaluation of the Effects of Antioxidants on UVA Photosensitization

Antioxidants were added to HBSS containing 1 µM riboflavin and 30 µM folic acid, or 1 µM riboflavin and 100 µM tryptophan, as noted. As antioxidants, 1 mM ascorbic acid (Sigma), 1 mM Trolox (Wako), 1 mM L-cysteine (Wako), and 1 mM L-histidine (Wako) were used. The amount of H_2_O_2_ generated in each solution after UVA irradiation with 3.6 J/cm_2_ was measured and compared.

### 4.12. H_2_O_2_ Scavenging Ability Assay

Each antioxidant was adjusted with HBSS so that the final concentration was 1 mM. Ten µL of 10 mM H_2_O_2_ was added to 1 mL of each antioxidant solution, and incubated for 5 min at room temperature. The amount of H_2_O_2_ in each anti-oxidant solution after incubation for 5 min was measured using the H_2_O_2_ assay.

### 4.13. Statistical Analysis 

Statistical analyses were performed on a minimum of three independent experiments using one-way analysis of the Dunnet II test. A *p*-value of <0.01 is regarded to be statistically significant.

## 5. Conclusions

Our study suggest that a low concentration of Riboflavin-derived photosensitization is elicited by different mechanisms depending on the coexisting vitamins and amino acids, and regulates cellular oxidative stress by producing H_2_O_2_ during UVA exposure.

## Figures and Tables

**Table 1:** Composition table of amino acids and vitamins contained in DMEM.

Amino Acids (in DMEM)	(g/L)	Vitamins (in DMEM)	(g/L)
A1: L-Arginine·HCl	0.084	V1: Choline chloride	0.004
A2: L-Cystine·2HCl	0.0626	V2: Folic acid	0.004
A3: Glycine	0.03	V3: myo-Inositol	0.0072
A4: L-Histidine·HCl·H_2_O	0.042	V4: Niacin amide	0.004
A5: H-Isoleucine	0.105	V5: D-Pantothenic acid·1/2Ca	0.004
A6: L-Leucine	0.105	V6: Pyridoxine·HCl	0.00404
A7: H-Lysine·HCl	0.146	V7: Riboflavin	0.0004
A8: L-Methionine	0.03	V8: Thiamine·HCl	0.004
A9: L-Phenylalanine	0.066		
A10: L-Serine	0.042		
A11: L-Threonine	0.095		
A12: L-Tryptophan	0.016		
A13: L-Tyrosine·2Na·2H_2_O	0.12037		
A14: L-Valine	0.094		

## Abbreviations

DMEM	Dulbecco’s modified Eagle’s medium
ROS	Reactive oxygen species
PBS	Phosphate Buffered Saline
HBSS	Hanks Balanced Salt Solution
GSH	Glutathione
H_2_O_2_	Hydrogen peroxide
UVA	Ultraviolet A
